# Yi-Qi-Ping-Chuan-Fang Reduces TSLP Elevation Caused by LPS + Poly(I:C) via Inhibiting TLR4/MYD88/NF-*κ*B Signaling Pathway

**DOI:** 10.1155/2017/3209407

**Published:** 2017-11-09

**Authors:** Minye Qu, Xiang Tao, Jian Ma

**Affiliations:** Department of Warm Disease, Basic Medical College, Nanjing University of Traditional Chinese Medicine, Nanjing, China

## Abstract

**Objective:**

To explore the correlation between Thymic Stromal Lymphopoietin (TSLP) and the Nuclear Factor- (NF-) *κ*B signaling pathways in bronchial epithelial cells and to clarify whether the traditional Chinese medicine formula Yi-Qi-Ping-Chuan-Fang (YQPC) reduces inflammation by inhibiting TSLP/NF-*κ*B signaling pathways.

**Methods:**

Cells were stimulated with LPS + Poly(I:C) and treated with YQPC. The expressions of TSLP and NF-*κ*B signaling pathways related proteins P65, I*κ*K, I*κ*Ba, P-P65, P-I*κ*K, P-I*κ*Ba were detected. The effects of NF-*κ*B upstream molecules, Toll-like receptors 3 and 4, myeloid differentiation primary response gene 88 (Myd88), TIR-domain-containing adapter-inducing interferon-*β* (TRIF), and downstream inflammatory cytokines, TNF-*α*, IL-1*β*, IL-6, and IL-8, were assessed.

**Results:**

The mRNA and protein expressions of TSLP were significantly increased after LPS + Poly(I:C) stimulation, the total protein I*κ*Ba and I*κ*K decreased (*P* < 0.05), and the phosphorylated protein P-P65, P-I*κ*K, and P-I*κ*B*α* increased. After YQPC treatment, the expression of TSLP, P-P65, P-I*κ*Ba, and P-I*κ*K was significantly inhibited (*P* < 0.05). The activation of TLR4 and MyD88 decreased, and release of IL-1*β*, IL-6, IL-8, and TNF-*α* reduced (*P* < 0.05).

**Conclusion:**

In summary, the expression of TSLP is activated by the NF-*κ*B signaling pathway. YQPC alleviated inflammation by inhibiting TSLP through regulating the NF-*κ*B activation and translocation.

## 1. Introduction

Bronchial asthma is a chronic inflammation of the airways affecting more than 300 million people worldwide, and its prevalence is increasing continuously [1, 2]. Airway inflammation is a basic pathological manifestation of asthma and is caused by the interaction of large numbers of inflammatory cells and cytokines [3]. The airway epithelial cells are considered the first physical barrier in the airway [4]. They participate in immune responses via the secretion of cytokines, chemokines, and inflammatory mediators when it first makes contact with allergens [5, 6].

TSLP is a cytokine released primarily by the epithelial cells that play an important role in the occurrence of asthma [7]. It combines with the DCs surface-specific receptor-TSLPr, drives naïve DCs (nDCs) to become mature DCs (mDCs), and strongly upregulates the expression of major histocompatibility complex (MHC) class II on mDCs. The TSLP-driven-mDCs then induce an abundant increase in naïve allogeneic CD4+ T cells, which differentiate into inflammatory TH2 cells, producing high levels of IL-5, IL-13, and IL-4 [7]. Excessive activation of TH2 results in an elevated IgE expression-mediated allergy [8]. Both IL-4 and IL-13 participate in the alteration of the IgE status in B cells [9].

Innate immune stimulating molecules, such as Toll-like receptor (TLR) ligands, are involved in the induction of TSLP [10]. Airway epithelial cells express functional TLRs, especially TLR 2–6 [11–13]. TLR3 ligand is recognized to induce TSLP expression in normal human bronchial epithelial cells [14]. The nonenzymatic mite allergen may stimulate an innate immune response through TLR4 by molecular mimicry of a lipid-recognition protein, MD-2 [15]. It was confirmed that functional TLR4 agonists can induce TSLP expression to trigger TH2-mediated allergic inflammation through TLR4-dependent innate immune pathways [16]. Therefore, in this study we selected TLR3 and TLR4 ligands as model agents to stimulate human bronchial epithelial cell line (16 HBE cells) to express TSLP.

Yi-Qi-Ping-Chuan-Fang (YQPC) is a classical Chinese medicinal formula in China that is composed of 10 traditional Chinese herbs: Herba Ephedrae (Mahuang), the dried rhizome of* Ephedra* plants; Asarum (Xixing), the dried root and rhizome of northern* Asarum* (Aristolochiaceae) plants; Fructus Schisandrae Chinensis (Wuweizi), the dried mature seeds of* Schisandra chinensis*; Fructus Perillae (Zisuzi), the dried ripe fruit of* Perilla*; *Scutellaria baicalensis* (Huangqin), the dried root of* Scutellaria* (Labiatae); Periostracum Cicadae (Chanyi), the exuviated shell of a cicada; Astragalus membranaceus (Huangqi), the dried root of Mongolian Astragalus membranaceus or membrane pod* Astragalus* root; large-headed atractylodes (Baizhu), the dried rhizome of Atractylodes (Compositae); Fangfeng, the root of the Umbelliferae plant; and licorice root (Shenggancao), the dried root and rhizome of the leguminous licorice plant.

YQPC has been used widely for the treatment of respiratory system diseases, including allergic asthma and allergic cough. The Chinese herbs Mahuang, Xixin, Wuweizi, and Gancao are the key components of Xiao-Qing-Long-Tang (XQLT) and have exhibited suppressive effects on airway inflammation and airway remodeling in animal models of asthma [17–19]. The Chinese herbs Huangqi, Baizhu, and Fangfeng make up another Chinese medicinal formula, Yu-Ping-Feng-San (YPFS). Recent studies have indicated that YPFS regulates the balance of TH1/TH2 cells in murine allergic airway disease via affecting the expression of the T cell receptor (TCR) and the MHC class II expression in a mouse model of asthma [20]. Huangqin and Zisuzi were found to have anti-inflammatory and immunity-regulating effects [20, 21]. However, research is limited on YQPC despite its wide clinical use. The action mechanism of YQPC is unknown.

The aim of this study was to investigate the mechanism of TSLP production in respiratory epithelial cells and to explore the clinical and immune-pharmacological effects of YQPC on TSLP production. We identified the optimal model that caused the 16 HBE cells to have a high expression of TSLP in vitro. We found that the NF-*κ*B signaling pathway was associated with TSLP expression in 16 HBE cells treated with LPS plus Poly(I:C). Furthermore, we demonstrated the role of YQPC on effectively reducing the expression of TSLP and several inflammatory factors through inhibition of the TLR4/MyD88/NF-*κ*B signaling pathways.

## 2. Materials and Methods

### 2.1. Preparation of YQPC

Ephedra, Asarum, and other 10 kinds of herbs were purchased from Guoyitang, Nanjing University of Chinese Medicine, Nanjing, China, and prepared with the proportion of 6 : 3 : 6 : 10 : 10 : 6 : 10 : 10 : 6 : 6. Herbal specimens of YQPC were obtained from the Collateral Disease Research Center at the Nanjing University of Chinese Medicine. The herbal mixture was immersed in 8 volumes of water (v/w) for 0.5 hours and then boiled for 1 hour. The extraction process was repeated twice and the extracts were combined. The mixture was filtered by a vacuum concentrator system (CH-9230; BUCHI Labortechnik, Flawil, Switzerland) at 60°C and dried thoroughly by lyophilization. The yield of YQPC was 52% and it was stored at 4°C in the form of a powder. The powder was dissolved in RPMI-1640 medium at the desired concentration and filtered through a nonfluorescent cotton filter (0.22 *μ*m pore size).

### 2.2. Cell Culture

The human bronchial epithelial cell line 16 HBE (provided by Shanghai Qiaodu Biological Technology Co., Ltd., Shanghai, China) was cultured in a RPMI-1640 medium (Wisent, Nanjing, China) supplemented with 10% fetal bovine serum (Wisent). The cells were incubated at 37°C in a 5% CO_2_ incubator (Thermo Scientific, Waltham, MA, USA).

### 2.3. Study Design and Drug Treatment

First, we determined the optimum times and concentrations for our subsequent studies. Cells were maintained in a serum-free RPMI-1640 medium for 24 hours, treated with several concentrations of LPS (Sigma Aldrich, Shanghai, China), Poly(I:C) (Sigma Aldrich), and LPS + Poly(I:C) for up to 24 hours. We selected the best modeling method by detecting the expression of TSLP.

Second, we investigated the direct correlation between TSLP expression and NF-*κ*B signaling pathway using BAY11-7082 [22] (Beyotime Biotechnology, Nantong, China) as a blocking agent for NF-*κ*B signaling. We divided the cells into three groups: (1) 10 ug/ml LPS + 10 ug/ml Poly(I:C) combined stimulated cells as positive control, (2) 10 ug/ml Poly(I:C) + 10 ug/ml LPS + 10 uM BAY11-7082 as inhibitor group, and (3) a serum-free RPMI-1640 medium as negative control. We evaluated the association between the expression of TSLP and the NF-*κ*B signaling pathway.

Finally, we treated LPS/Poly(I:C) stimulated cells with Chinese herbal formula YQPC. One group of cells was cultured with a serum-free RPMI-1640 medium as negative control and three groups were treated with LPS + Poly(I:C) and a concentration gradient of YQPC (2 ug/ml, 3 ug/ml, and 4 ug/ml). Treatment group without YQPC was used as positive control. We examined the inhibitory effect of YQPC on TSLP/NF-*κ*B signaling pathway and evaluated the regulatory effects of YQPC on NF-*κ*B upstream signal molecules, MyD88, TRIF, TLR3, and TLR4, and downstream inflammatory factors, IL-1*β*, IL-6, IL-8, and TNF-*α*.

### 2.4. Cell Viability Assay

Cell viability was detected by the MTT assay. Cells were seeded in 96-well plates at a density of 2 × 10^4^ cells/well in a volume of 200 uL/well. When the cells reached 70% confluence, they were, respectively, pretreated by several concentrations of YQPC (0–5 ug/ml) and YQPC + LPS/Poly(I:C) for 24 hours (all experiments were performed in triplicate wells for each condition). Following the treatments, 20 ul of MTT (0.5 mg/ml, Sigma Aldrich) was incubated with the pretreated cells for 4 hours at 37°C. After incubation, 150 ul of DMSO was added to the cells for 15 minutes at room temperature, and the absorbance was measured at 490 nm using a microplate reader (Bio-Rad Laboratories, Hercules, CA, USA). The percentage of living cells was evaluated in comparison to the untreated control cells.

### 2.5. Quantitative Real-Time PCR

In order to select the best modeling method, cells were harvested after treatment with different concentrations of LPS or Poly(I:C) at different times. The expression of TSLP mRNA was detected by quantitative real-time polymerase chain reaction (qRT-PCR). To evaluate the effects of YQPC on TSLP expression and activation of TLR signaling pathway induced by LPS + Poly(I:C), cells were treated with LPS + Poly(I:C) (10 ug/ml, 10 ug/ml) and incubated with different concentrations (2 ug/ml, 3 ug/ml, and 4 ug/ml) of YQPC for 24 hours. LPS + Poly(I:C) treated cells with no YQPC were used as positive control. The cells were then harvested and the mRNA levels of expression of TSLP, TLR3, TLR4, Myd88, and TRIF were measured by qRT-PCR. The specific primers of TSLP, TLR3, TLR4, MyD88, TRIF, and *β*-actin (the reference gene) are listed in Table 1. The total RNA was extracted via the TRIzol® Max™ Bacterial RNA Isolation Kit (Invitrogen, Carlsbad, CA, USA). The purity and concentrations of RNA were determined using a NanoDrop ND-1000 instrument (Thermo Scientific), and the samples were stored at −80°C. cDNA synthesis from the total RNA (0.5 ug) was performed using PrimeScript™ RT Master Mix (Takara Biotechnology, Dalian, China). The Q-PCR reactions (final volume 25 uL) contained 500 ng cDNA and 10 uM of the forward and reverse primers; we used the TransStart Green Q-PCR SuperMix (TransGen Biotech, Beijing, China). The reaction mixtures were utilized after being exposed to the following conditions: initial denaturation at 95°C for 10 minutes followed by 45 cycles at 95°C for 30 seconds, 60°C for 1 minute, and 72°C for 45 seconds. PCR experiments were performed in triplicate. The amplification data measured by fluorescence were collected in real time and analyzed by the QuantStudioTM Real-Time PCR system (Thermo Scientific) using the 2^−ΔΔCt^ method.

### 2.6. Western Blot

Cells were harvested after treatment with LPS + Poly(I:C) and LPS + Poly(I:C)+YQPC (concentrations ranged from 0 to 4 ug/ml). The cells were rinsed thrice with ice-cold Phosphate Buffered Saline (PBS) and lysed in a RIPA buffer (Beyotime Biotechnology) containing 1 ul/100 ul protease inhibitors and a phosphatase inhibitor mixture (Vazyme Biotech, Nanjing, China). The samples were dissolved under low temperatures for 10 minutes, and the lysates were centrifuged at 12,000*g* in a microcentrifuge (Thermo Scientific) for 20 minutes at 4°C. The protein concentration was determined with the Pierce™ BCA Protein Assay Kit (Thermo Scientific). Samples containing 25 ug of protein were mixed with 5 × SDS sample buffer and boiled for 10 minutes, separated by SDS-PAGE with 8–12% acrylamide gel, and transferred to as a PVDF membrane (Millipore, Bedford, MA, USA). The membrane was blocked with Tris-buffered saline (TBS) containing 0.05% Tween 20 and 5% BSA (Beyotime Biotechnology) at room temperature for 2 hours. After blocking, membranes were incubated overnight at 4°C with the primary antibody TSLP antibody (1 : 1000, ab47943, Abcam, Cambridge, UK), Phospho-NF-*κ*B P65 (Ser536) antibody (1 : 1000, #3031, Cell Signaling, Boston, MA, USA), NF-*κ*B antibody (1 : 1000, #3032, Cell Signaling), I*κ*K alpha antibody (1 : 1000, #2682, Cell Signaling), Anti-I*κ*K alpha (phospho S176) antibody (1 : 1000, ab138426, Abcam), I*κ*B*α* antibody (1 : 1000; Santa Cruz Biotechnology, Santa Cruz, CA, USA), Phospho-I*κ*B*α* (Ser32/36) antibody (1 : 1000, #9246, Cell Signaling), and *β*-actin antibody (1 : 1000, Abclone, Wuhan, China). After that, the membranes were washed with TBS 1 × 0.1% Tween 20 three times for 10 minutes each time and then incubated with the secondary HRP-conjugated antibody, goat anti-rabbit IgG-HRP (1 : 5000; Santa Cruz Biotechnology), at room temperature for 1.5 hours. The protein immunoreactivity was revealed with the ECL system (Millipore) by the Molecular Imager® ChemiDoc™ XRS System (Bio-Rad Laboratories).

### 2.7. Immunofluorescence

Cells were treated with 10 ug/ml LPS + 10 ug/ml Poly(I:C) and intervened with 0–4 ug/ml YQPC for 20 minutes and 12 hours. Then the cells were washed in ice-cold PBS three times for 3 minutes each time. The cells were fixed in 4% paraformaldehyde at room temperature for 15 minutes, washed with PBS for 30 minutes, and permeabilized with 0.5% Triton X-100 in PBS for 20 minutes. The cells were rinsed with PBS before being blocked with 10% goat serum (Solarbio Life Sciences, Beijing, China) in PBS for 1 hour at 37°C. We then subsequently incubated the cells with the NF-*κ*B antibody (1 : 400, #3032, Cell Signaling) and TSLP antibody (1 : 200, ab47943, Abcam) at 4°C overnight. The cells were washed with PBS three times and then incubated with the second antibody using the immunofluorescence staining kits, anti-rabbit kFluor488, and anti-rabbit kFluor555 (Keygen Biotech, Nanjing, China), at room temperature for 1 hour, respectively. The cells were then washed and dyed with the Hoechst Staining Kit (Beyotime Biotechnology) for 10 minutes. We viewed the labeled sections with fluorescence confocal microscopy (Olympus, Tokyo, Japan).

### 2.8. Enzyme-Linked Immunosorbent Assay

Cells were treated with 10 ug/ml LPS + 10 ug/ml Poly(I:C) and intervened with 0–4 ug/ml YQPC for 24 hours. Concentrations of TNF-*α*, IL-1*β*, TGF-*β*, IL-6, and IL-8 in the cell culture supernatant were determined by enzyme-linked immunosorbent assay (ELISA) using Quantikine ELISA kits (R&D Systems, Minneapolis, MN, USA) according to the manufacturer's instructions. The absorbance values were read at 450 nm, and we compared their optical densities with a standard curve to determine the concentration. The results were expressed as pg/ml of the culture supernatants.

### 2.9. Statistical Analysis

All data were expressed as mean ± standard error of mean (SEM) and analyzed using SPSS 18.0 statistical software (SPSS Inc., Chicago, IL, USA). A one-way analysis of variance was used in multiple groups to assess statistical significance. The *P* value < 0.05 was considered to be meaningful, and the *P* value < 0.01 was considered to be statistically significant.

## 3. Results

### 3.1. LPS Associated with Poly(I:C) Increases the Expression of TSLP in 16 HBE Cells

**Figure 1:**
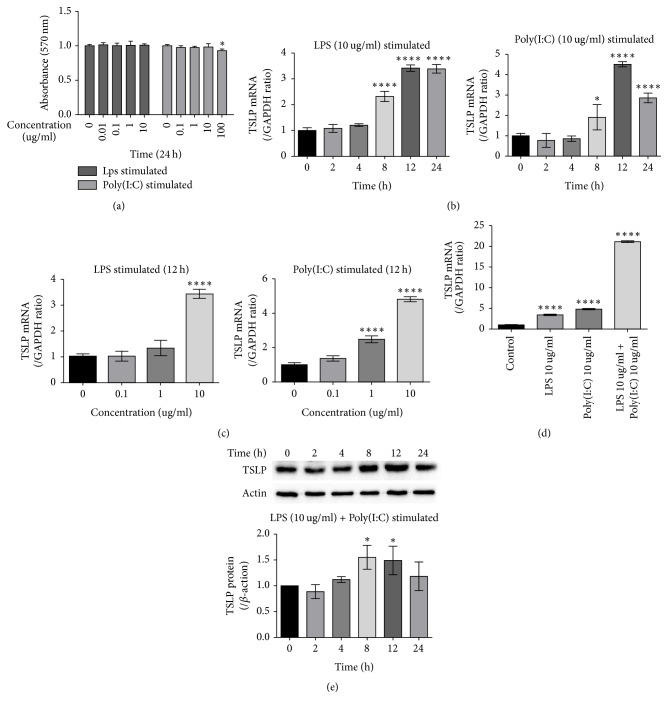
*Effects of LPS and Poly(i:c) on TSLP expression in 16 HBE cells*. (a) Effect of LPS and Poly(I:C) on 16 HBE cell proliferation for 24 h. (b) 10 ug/ml LPS and 10 ug/ml Poly(I:C), respectively, stimulated 16 HBE cells, 0 to 24 h. (c) 0–10 ug/ml LPS and 0–10 ug/ml Poly(I:C), respectively, stimulated 16 HBE cells for 12 h. (d) 16 HBE cells were stimulated with LPS, Poly(I:C), and LPS + Poly(I:C) for 12 h. (e) 16 HBE cells were stimulated with LPS + Poly(I:C) 0 to 24 h. ^*∗*^*P* < 0.05 and ^*∗∗∗∗*^*P* < 0.0001.

We explored the optimum time and concentration of the reagent that caused the TSLP expression to become elevated. As shown in Figure 1(a), both the LPS or Poly(I:C) concentrations between 0 and 10 ug/ml showed no definite effect on cell viability. To assess the effect of LPS or Poly(I:C) on the TSLP expression in 16 HBE, we measured the expression of the TSLP mRNA in the range of 0 to 24 hours in LPS 10 ug/ml and Poly(I:C) 10 ug/ml. Both the LPS-mediated and Poly(I:C)-mediated expressions of the TSLP mRNA were increased at 8 hours (*P* < 0.0001, *P* < 0.05) and peaked at 12 hours (*P* < 0.0001, *P* < 0.0001). The difference was that the expression of mRNA remained high after 24 hours of LPS stimulation, whereas the expression of Poly(I:C) stimulated cells was reduced under the same conditions (Figure 1(b)). We speculated that the most reasonable time for the cell stimulation was 12 hours. To screen for the optimum concentration, we used 0 ug/ml, 0.1 ug/ml, 1 ug/ml, and 10 ug/ml LPS and the corresponding concentration of Poly(I:C) stimulated cells for 12 hours. We found that 10 ug/ml LPS and 10 ug/ml Poly(I:C) resulted in a significant increase in TSLP mRNA (*P* < 0.0001, *P* < 0.0001) (Figure 1(c)). We investigated whether the LPS and Poly(I:C) combined stimulation could induce a pronounced TSLP expression. We combined 10 ug/ml LPS and 10 ug/ml Poly(I:C) stimulated 16 HBE for 12 hours for comparison with the single stimulation group and found that the expression of TSLP mRNA in the combination group was significantly higher than that in the single stimulation group (*P* < 0.0001) (Figure 1(d)). Then we examined the expression of LPS and Poly(I:C) joint-mediated TSLP proteins from 0 to 24 hours. As shown in Figure 1(e), the expression of the TSLP protein was elevated to a peak between 8 and 12 hours (*P* < 0.05, *P* < 0.05) and had decreased by 24 hours. All of these data support 10 ug/ml LPS plus 10 ug/ml Poly(I:C) stimulated cells for 12 hours to cause increased TSLP gene and protein expression.

### 3.2. TSLP Expression Is Related to the Activation of NF-*κ*B Pathway

**Figure 2:**
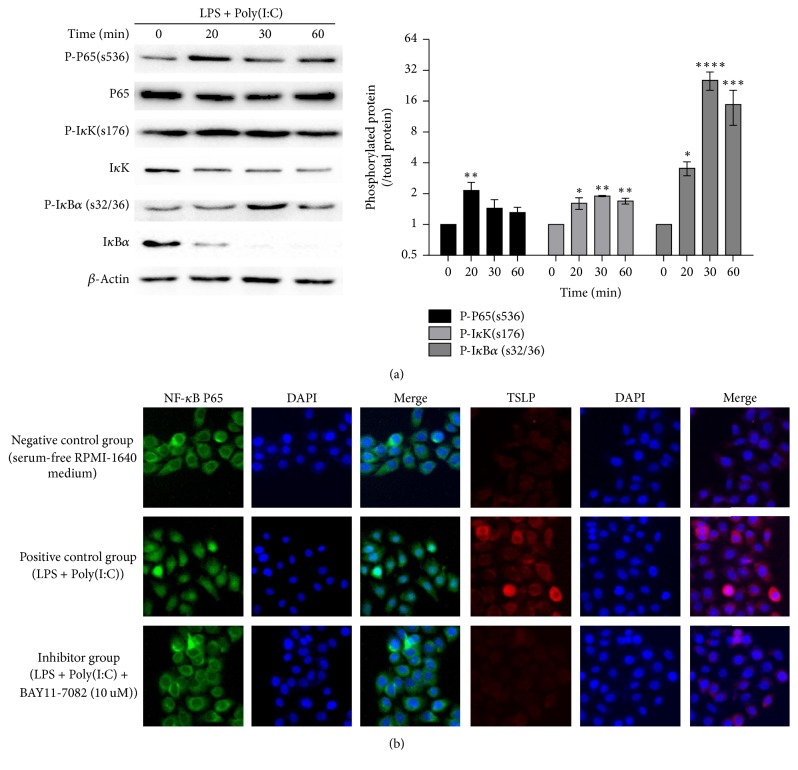
*LPS + Poly(I:C) activated the NF-κB signaling pathway to promote TSLP expression*. (a) NF-*κ*B signaling pathway-related protein expression activated by LPS + Poly(I:C) in 0–60 minutes was tested by western blot. ^*∗*^*P* < 0.05, ^*∗∗*^*P* < 0.01, ^*∗∗∗*^*P* < 0.001, and ^*∗∗∗∗*^*P* < 0.0001. (b) NF-*κ*B translocation and TSLP expression in 16 HBE cells were examined by immunofluorescence assay. *n*  =  3. All the experiments were performed in triplicate.

We investigated the expression of the NF-*κ*B activation pathway under LPS/Poly(I:C) stimulation.  Figure 2 showed that the expression of the total amount of proteins P65, I*κ*K, and I*κ*B*α* decreased, whereas the expression of P-P65, P-I*κ*B*α*, and P-I*κ*K increased after stimulation. Compared to the corresponding total proteins, the expression of P-P65, P-I*κ*B*α*, and P-I*κ*K reached peaks at 20 minutes, 30 minutes, and 30 minutes, respectively (*P* < 0.01, *P* < 0.01, and *P* < 0.0001).

We next attempted to illustrate whether the NF-*κ*B pathway is directly related to the expression of TSLP. Immunofluorescence revealed that NF-*κ*B P65 was predominantly present in the nucleus with the treatment of Poly(I:C) + LPS, whereas the negative control group and the inhibitor group showed that P65 was mainly distributed around the nucleus (Figure 2(b)). However, the expression of TSLP showed a significant increase after Poly(I:C) + LPS stimulation, but it was significantly lower in the NF-*κ*B inhibitor group, and no significant difference was found with the negative control group.

### 3.3. Evaluation of YQPC Cytotoxicity in 16 HBE Cells

**Figure 3:**
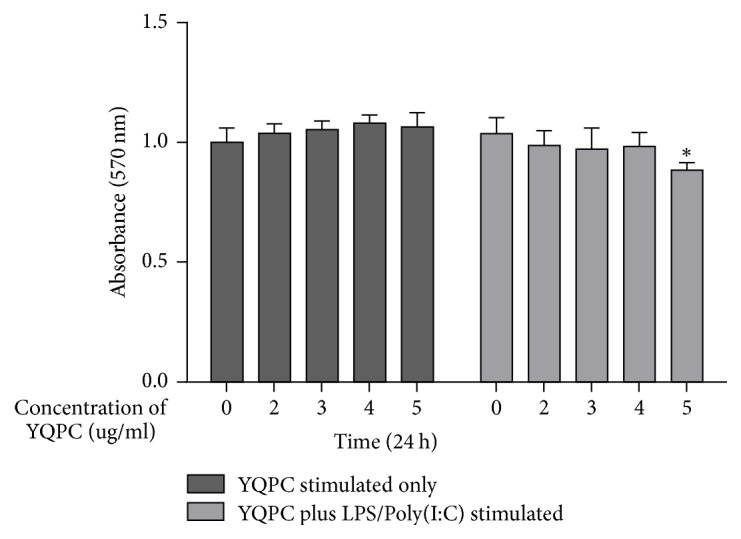
*Effect of YQPC on 16 HBE cell proliferation*. Cells were treated with several concentrations of YQPC (0–5 ug/ml) and YQPC + LPS/Poly(I:C) for 24 h to assess cell viability using MTT assay. ^*∗*^*P* < 0.05.

We then performed an experiment to avoid the effects of drug toxicity on the cells and to screen for the safest concentration range of the drug.  Figure 3 showed that YQPC had no significant effect on 16 HBE at the concentrations between 0 and 5 ug/ml. To exclude YQPC + LPS/Poly(I:C) which may increase cytotoxicity, cells were treated with concentrations of 0–5 ug/ml YQPC + LPS (10 ug/ml) + Poly(I:C) (10 ug/ml) for 24 hours and compared with the control. We found that cell viability was reduced after LPS + Poly(I:C) + 5 ug/ml YQPC treatment (*P* < 0.05). There was no effect on cell viability when YQPC was at concentrations between 0 and 4 ug/ml coupled with LPS + Poly(I:C). So we can eliminate the possibility that YQPC cytotoxicity would affect the protein expression at a concentration of 0–4 ug/ml in subsequent experiments.

### 3.4. YQPC Inhibits TSLP Expression by Activating of NF-*κ*B Signaling Pathway

**Figure 4:**
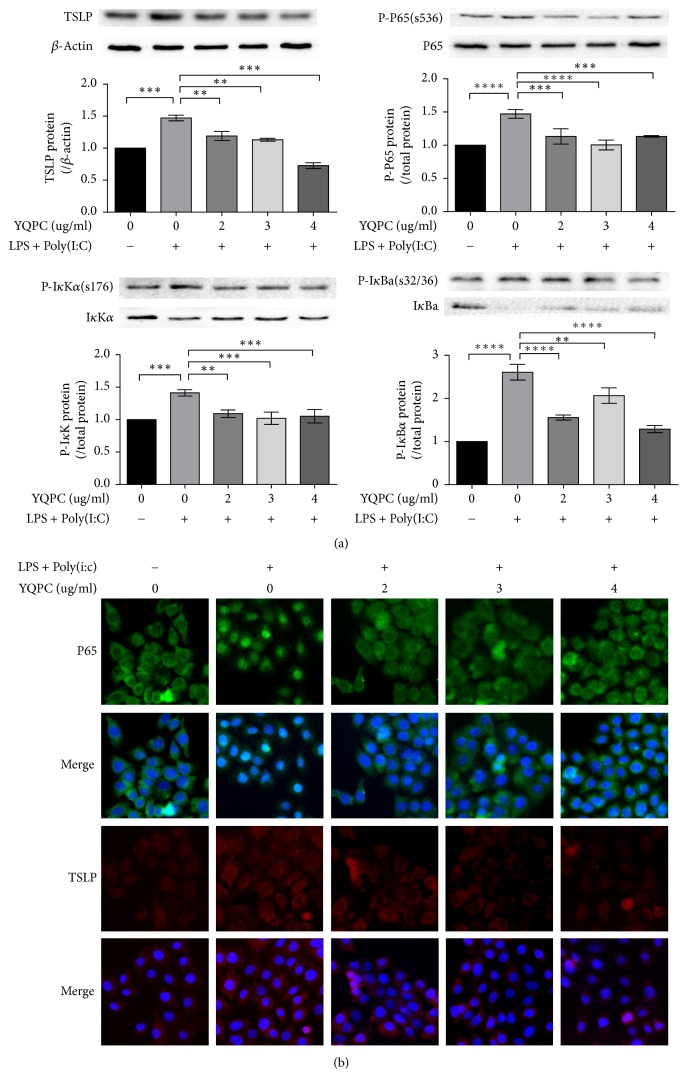
*YQPC reduced the expression of TSLP by inhibiting the NF-κB signaling pathway*. (a) The expression of TSLP, P65, I*κ*B*α*, I*κ*K*α*, P-P65, P-I*κ*B*α*, and P-I*κ*K*α* in YQPC pretreated 16 HBE cells was tested by western blot. ^*∗∗*^*P* < 0.01,^*∗∗∗*^*P* < 0.001, and ^*∗∗∗∗*^*P* < 0.0001. (b) The effect of YQPC on TSLP expression and NF-*κ*B nuclear translocation was examined by immunofluorescence assay. *n*  =  3. All the experiments were performed in triplicate.

We explored the effect of YQPC on TSLP and the NF-*κ*B signaling pathways in 16 HBE. We harvested cells at different time points (as described in Figure 2) to detect the expression of TSLP and the NF-*κ*B pathway-related proteins.  Figure 4(a) showed that the TSLP expression increased significantly (*P* < 0.001) in the positive control group and was downregulated after the YQPC treatment, especially at the concentration of 4 ug/ml (*P* < 0.0001). The expression of phosphorylated protein P-P65, P-I*κ*K, and P-I*κ*B*α* was increased when stimulated with LPS + Poly(I:C) without the YQPC intervention (*P* < 0.0001, *P* < 0.001, and *P* < 0.0001). The medicated groups all effectively reduced the expression of P-P65, P-I*κ*K, and P-I*κ*B*α* in the treated cells.

We then observed the YQPC suppression of the TSLP expression and P65 nuclear translocation by immunofluorescence. As shown in Figure 4(b), NF-*κ*B P65 nuclear translocation was shown after LPS + Poly(I:C) treatment. The medicated groups inhibited the movement of P65 into the nucleus, and the extent of inhibition was generally proportional to the treatment concentrations. In addition, the expression of TSLP was increased after LPS + Poly(I:C) stimulation and decreased in each YQPC treatment group.

### 3.5. YQPC Inhibits the Expression of TLR4 and MyD88

**Figure 5:**
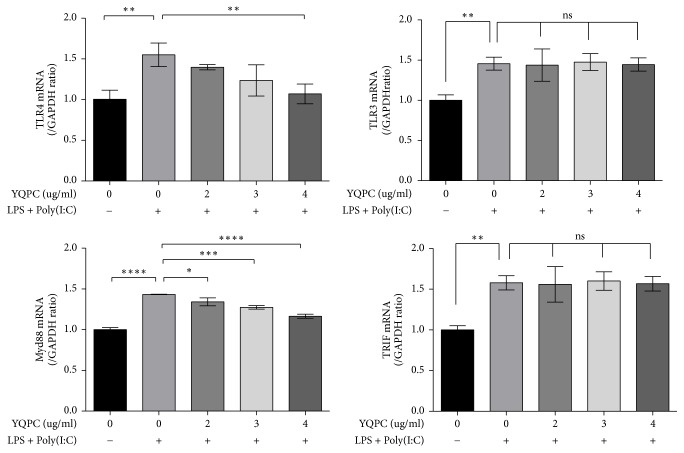
*The effect of YQPC on the expression of upstream molecules in the NF-κB signaling pathway*. The expression of TLR3 mRNA, TLR4 mRNA, and Myd88 mRNA after YQPC treatment was measured by Q-PCR. ^*∗*^*P* < 0.05, ^*∗∗*^*P* < 0.01, ^*∗∗∗*^*P* < 0.001, and ^*∗∗∗∗*^*P* < 0.0001.

We investigated the effect of YQPC on the expression of upstream molecules in the NF-*κ*B signaling pathway. As shown in Figure 5, the expression of TLR3 mRNA, TLR4 mRNA, MyD88 mRNA, and TRIF mRNA increased significantly after LPS + Poly(I:C) stimulation (*P* < 0.01, *P* < 0.01, *P* < 0.0001, and *P* < 0.01). YQPC slightly inhibited the expression of Poly(I:C)/LPS-activated TLR4 and MyD88 and enhanced the inhibitory effect as the concentration increased. However, its inhibition of TLR3 and TRIF was not obvious.

We verified that YQPC had a suppressive effect on the LPS-related immune response but not the Poly(I:C)-related response. Cells were stimulated with Poly(I:C) and LPS separately and compared with their respective Chinese medicinal formula intervention group. The results showed that the expression of the TSLP mRNA increased after LPS stimulation and significantly decreased after the YQPC intervention (*P* < 0.0001). However, regardless of whether the sample received the YQPC intervention, the expression of TSLP mRNA did not decrease after the Poly(I:C) stimulation (Supplement 1b in Supplementary Material available online at https://doi.org/10.1155/2017/3209407). The expression of the protein P-P65 also revealed an inhibitory effect of YQPC on LPS-induced NF-*κ*B activation (Supplement 1a).

### 3.6. YQPC Inhibits the Release of NF-*κ*B Activation Related Inflammatory Cytokines

**Figure 6:**
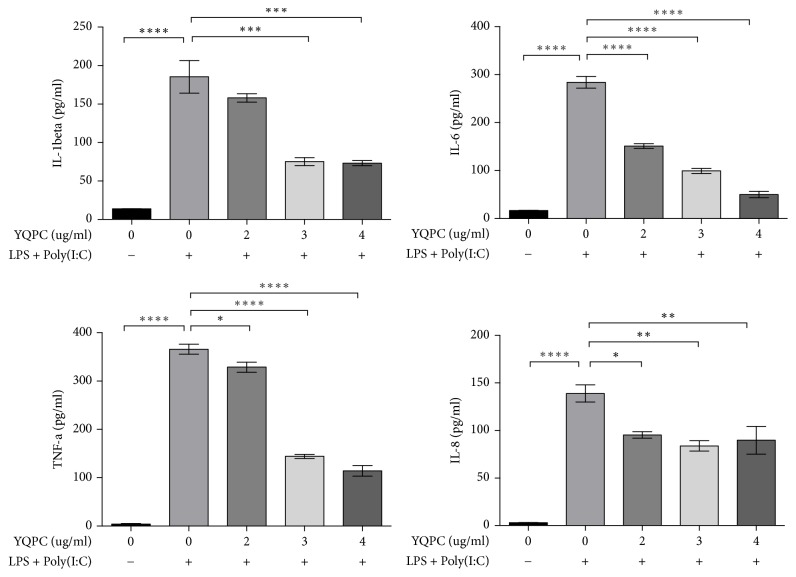
*The effect of YQPC on the expression of downstream molecules in the NF-κB signaling pathway*. The expression of IL-1*β*, IL-6, TNF-*α*, and IL-8 after YQPC treatment was examined by ELISA assay. ^*∗*^*P* < 0.05, ^*∗∗*^*P* < 0.01, ^*∗∗∗*^*P* < 0.001, and ^*∗∗∗∗*^*P* < 0.0001.

We investigated the effect of YQPC on the expression of downstream molecules in the NF-*κ*B signaling pathway. We found that LPS + Poly(I:C) resulted in 16 HBE cells that produced high levels of IL-6, IL-8, TNF-*α*, and IL-1*β* (*P* < 0.0001, *P* < 0.0001, *P* < 0.0001, and *P* < 0.0001). In contrast, cells treated with YQPC produced a lower level of these inflammatory cytokines, and the inhibitory effect was enhanced with the increase in the drug concentration (Figure 6).

## 4. Discussion 

In this study, we explored the correlation between the NF-*κ*B pathway and TSLP expression and the inhibition by a traditional Chinese medicinal formula, YQPC. We showed that YQPC inhibited the TSLP expression by attenuating the TLR4/MyD88/NF-*κ*B signaling pathways. YQPC can significantly reduce the expression of the inflammatory cytokines IL-6, IL-8, TNF-*α*, and IL-1*β*.

The airway epithelium acts as a physical barrier to protect the trachea against bacteria, pathogens, and other antigens invading the bronchus and making contact with the immune system to prime abnormal immune responses [4]. TSLP, secreted by the bronchial epithelial cells [23], is the main switch of allergic disease. When allergens or pathogens are inhaled, DCs are activated to prime CD4+ TH2 cell development [24] to induce the TH2 atopic immune response characteristic of asthma. More and more researches suggested that it is the most important target interfering with the initial phase of allergic diseases [25–27]. In this study, we used normal bronchial epithelial cells, and the expression of TSLP is low in the physiological state. In order to simulate the expression of TSLP in pathological state, we screened the method of modeling using LPS and Poly(I:C). The results showed that the expression of TSLP mRNA in LPS + Poly(I:C) stimulated group was significantly higher than that in the single stimulation group. Based on subsequent experiments, we found that the expression of TSLP was directly related to the activation of the NF-*κ*B signaling pathway. LPS and Poly(I:c) are Toll-like receptor agonists, through a series of signal molecules to activate NF-*κ*B signal pathway [28, 29]. LPS + Poly(I:C) plays a synergistic role in stimulating cells to induce TSLP expression. Another interesting finding is that the expression of the TSLP gene reached a peak at 12 hours, whereas the expression of the protein continued to peak at 8 to 12 hours after LPS + Poly(I:C) stimulation, because in LPS and Poly(I:C) single stimulation groups the expression of the TSLP gene was significantly increased at 8 hours. So the time for gene starting transcription should be earlier than 8 hours (between 4 and 8 hours after stimulation). Moreover, changes in protein levels occur almost simultaneously with changes in gene levels. Protein translation is fast, sometimes overtranslated, and proteins have longer half-lives than genes.

Because airway inflammation is the characteristic feature of asthma, NF-*κ*B, a proinflammatory transcription factor, and its complex modulation are found to play a central role in inflammatory airway disease [30]. The latest research reports that the inhibition of NF-*κ*B is a new target for the treatment of asthma [30]. We theorized that there must be an inherent relationship between TSLP and NF-*κ*B in the bronchial epithelial cells. In the classical NF-*κ*B activation pathway, the NF-*κ*B/Rela dimer is present in the cytoplasm in an inactive state. When stimulated by cytokines or microorganisms, IKB kinase (IKK*α*/*β*complex) is activated, promoting IKBs phosphorylation and eventual ubiquitination and degradation. The degradation of IKBs leads to the transfer of P50 and P65 to the nucleus [31]. Therefore, we selected I*κ*K*α*, I*κ*B*α*, P65, P-I*κ*K, P-I*κ*B*α*, and P-P65 as the NF-KB signal pathway detection indicators. We chose the time points to observe the activation of NF-*κ*B between 0 and 60 minutes. Surprisingly, the NF-*κ*B signaling pathway was activated after LPS/Poly(I:C) stimulation, much earlier than the TSLP expression. Based on previous experiments, the expression of TSLP protein reached a peak at 12 hours. The expression of NF-*κ*B-associated phosphorylated protein P-P65 reached a peak at 20 minutes, which means that the nuclear transfer occurred at this point in time. So we cultured the cells for 20 minutes and 12 hours, respectively, and added the NF-*κ*B inhibitor BAY11-7082 to confirm whether the expression of TSLP was activated by the NF-*κ*B signaling pathway. We found that the expression of TSLP was weakened with the inhibition of the NF-*κ*B signal pathway. These results suggest that the NF-*κ*B signaling pathway plays an important role in the production of TSLP in 16 HBE cells.

Although YQPC is widely used in the effective treatment of allergic diseases, the research is not sufficient. YQPC components Yupingfengsan (YPFS) and Xiaoqinglongtang (XQLT) had been researched a lot, but these are rarely used alone in actual clinical settings. Many studies have focused on correcting the imbalance of TH1/TH2 in allergic diseases, and the role of these medicines is clear [32, 33]. However, if we view the TH2 inhibitory effect from the TSLP-DC-TH2 axis [7], their specific targets are unclear. We tested whether YQPC could prevent the occurrence of an allergy by suppressing the expression of TSLP upstream of the TSLP-DC-TH2 axis. Therefore, we used different concentrations of YQPC on the stimulated cells. The TSLP expression reached its peak time at 12 hours. Compared with the positive control group, the herbal groups showed different degrees of reduction. In previous studies, we found that the NF-*κ*B signaling pathway was involved in the expression of TSLP, so we examined the effect of YQPC on NF-*κ*B at different peak expression times. The result is that the effect of YQPC on the NF-*κ*B signaling pathway is inhibitory. On the basis of these results, we can conclude that YQPC reduces the expression of TSLP by inhibiting the NF-*κ*B signaling pathway. We did not perform further screening of the active ingredients of this traditional Chinese medicine, but we do intend to explore them in future studies.

Toll-like receptors (TLRs) are a subset of pattern recognition receptors (PRRs) that play key roles in the innate immune system by recognizing several pathogen-associated molecular patterns (PAMPs) derived from various microbes. TLRs signal through the recruitment of specific adaptor molecules, leading to activation of the transcription factors NF-*κ*B [34]. LPS and Poly(I:C) are agonists of TLR4 and TLR3, respectively, and MyD88 and TRIF are adaptors molecules in response to activation of TLRs [28, 34]. TLR4 uses MyD88 and/or TRIF-dependent pathways to activate NF-*κ*B pathways [35]. TLR4 and MyD88 signaling results in the recruitment of downstream signaling molecules IRAK-4 and IRAK-1, followed by TRAF6 activation. The activated TRAF6 has ubiquitin ligase E3 activity and degrades IKKs, leading to the degradation of I*κ*B, releasing NF-*κ*B to translocate into the nucleus [36]. In addition, TLR4 recruits TRIF indirectly with TRIF-related adaptor molecule (TRAM). TRIF binds TRAF6 through the N-terminal T6BM structure and mediates NF-*κ*B activation. Unlike TLR4, TLR3 directly interacts with TRIF. When activated by Poly(I:C), TLR3 induces cytokine production. Poly(I:C) can also induce activation of NF-*κ*B and mitogen-activated protein (MAP) kinases, independent of MyD88 [28, 29]. Previous studies showed that YQPC inhibits NF-*κ*B signaling pathway. To demonstrate whether this inhibition is related to its upstream Toll-like receptors, we tested TLR3, TLR4, MyD88, and TRIF. We found that YQPC attenuated the expression of TLR4 and MyD88, but it had no effect on TLR3 and TRIF, which indicated that YQPC had no inhibitory effect on the Poly(I:C)-activated NF-*κ*B pathway. To demonstrate this conclusion, we compared the expression of the downstream product of NF-*κ*B in either LPS or Poly(I:C) stimulated groups and a single stimulated plus YQPC intervention group. The results were consistent: the inhibitory effect of YQPC was only seen in the LPS stimulation group. Therefore, we speculate that YQPC inhibits NF-*κ*B activation by attenuating the LPS-activated TLR4/MyD88 signaling pathway.

Bronchial asthma is a chronic airway inflammation that involves many inflammatory cytokines. NF-*κ*B, as an inflammatory transcription factor, interacts with cytokines to participate in the development of inflammation [37]. IL-1, TNF-a, and IL-6 are considered to be expressed in most types of inflammation, especially asthma [38]. IL-1*β* amplifies innate immune responses and promotes the expression of various NF-*κ*B-dependent cytokines and chemokines [39]. NF-*κ*B activation can produce a variety of products, such as TNF-a, cox-2, and NO [40]. IL-8, which is involved in neutrophil recruitment, is synthesized and secreted under the stimulation of IL-1*β*, TNF-*α*, and LPS [41]. In this study, we examined IL-6, IL-8, TNF-*α*, and IL-1*β* to detect the inhibitory effect of YQPC on inflammation. The results showed that YQPC had a weakening effect on the expression of these inflammatory factors. We concluded that YQPC inhibited the release of the NF-*κ*B-activation-related inflammatory cytokines.

In summary, YQPC alleviated allergic inflammation by inhibiting TSLP through regulating the NF-*κ*B activation and translocation. However, there were several limitations to the present study. Because the composition of this traditional Chinese medicine formula is complex, we cannot prove that the regulatory effect is the role of a single herbal medicine or the result of herbal synergies. In a future study, we will separate and recombine the parts of the formula to identify the specific components of suppression by screening the active components from YQPC. Moreover, we will study YQPC for DC and T cell influence to define the role of YQPC for the entire TSLP-DC-TH axis. These studies might provide a theoretical basis for YQPC in the clinical treatment of asthma.

## 5. Conclusion 

**Figure 7:**
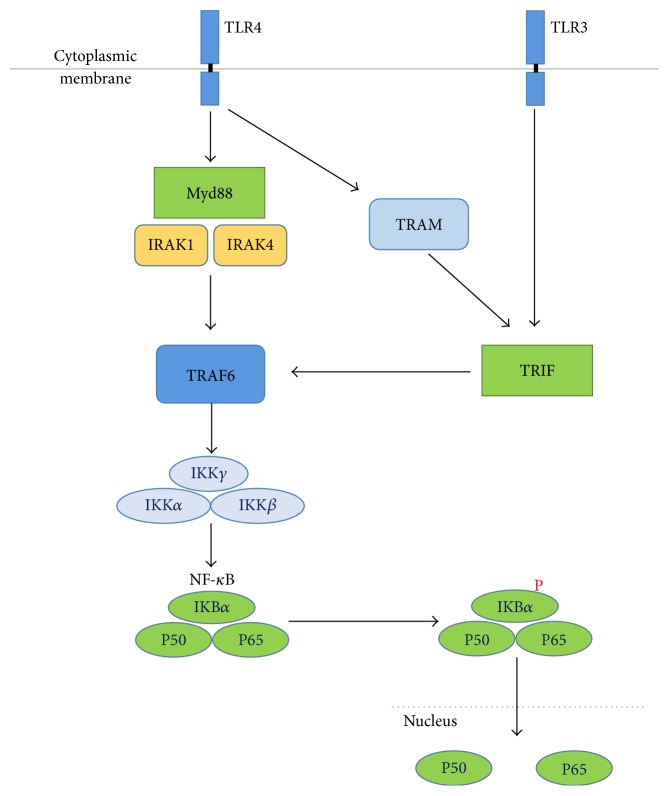
The NF-*κ*B signaling pathway is activated by TLR3/TLR4.

This study demonstrates that YQPC could attenuate the immune response induced by LPS + Poly(I:C). The protective effect of YQPC is shown to result from inhibiting the proinflammatory cytokine TSLP and is also associated with the down-regulation of TLR4/MyD88/NF-*κ*B signaling pathway (Figure 7). Our findings suggest that YQPC might act as a potential therapeutic agent for the treatment of asthma.

## Supplementary Material

Supplement 1: YQPC inhibits TSLP expression by attenuating LPS-activated NF-κB signaling pathway.

## Figures and Tables

**Table 1:** The primer sequences used in this study.

Primer names	Sequence direction (5′→3′)
TSLP F	CCCAGGCTATTCGGAAACTCAG
TSLP R	CGCCACAATCCTTGTAATTGTG
TLR3 F	CCGCCAACTTCACAAGGTAT
TLR3 R	AGCTCATTGTGCTGGAGGTT
TLR4 F	TGGTCAGTGTGCTTGTGGTA
TLR4 R	GTTTCTCACCCAGTCCTCATT
MyD88 F	GCACATGGGCACATACAGAC
MyD88 R	TAGCTGTTCCTGGGAGCTGT
TRIF F	CAAGCCGTGCCCACCTACT
TRIF R	TGTTCCGATGATGATTCCAG
GAPDH F	GTGGACATCCGCAAAGAC
GAPDH R	GAAAGGGTGTAACGCAACT
