# Supplementary material for: Yi-Qi-Ping-Chuan-Fang Reduces TSLP Elevation Caused by LPS + Poly(I:C) via Inhibiting TLR4/MYD88/NF-κB Signaling Pathway
Source: Evid Based Complement Alternat Med. 2017 Nov 9;2017:3209407. doi: 10.1155/2017/3209407 (PMC5700476; doi:10.1155/2017/3209407)
Supplement: Supplementary file 1 — Supplement 1: YQPC inhibits TSLP expression by attenuating LPS-activated NF-κB signaling pathway. [file 3209407.f1.docx]

**Supplement 1. YQPC inhibits TSLP expression by attenuating LPS-activated NF-κB signaling pathway.**

To demonstrate that the inhibition of LPS + Poly (I: C) -related immune responses by YQPC is due to the inhibition of LPS or Poly (i: c). Cells were stimulated with Poly(I:C) and LPS separately and compared with their respective Chinese medicinal formula intervention group. The results showed that the expression of the TSLP mRNA increased after LPS stimulation and significantly decreased after the YQPC intervention (P<0.0001). However, regardless of whether the sample received the YQPC intervention, the expression of TSLP mRNA did not decrease after the Poly(I:C) stimulation (Supplement 1b). The expression of the protein P-P65 also revealed an inhibitory effect of YQPC on LPS-induced NF-κB activation (Supplement 1a). Based on the above experiment, we conclude that YQPC had a suppressive effect on the LPS-related immune response but not the Poly(I:C)-related response.

**Supplement 1. YQPC inhibits TSLP expression by attenuating LPS-activated NF-κB signaling pathway.**

The expression of downstream product of NF-κB in either LPS or Poly(I:C) stimulated groups and a single stimulated plus YQPC intervention group were compared by western blot and Q-PCR. **** p<0.0001

**b**


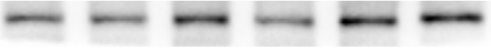

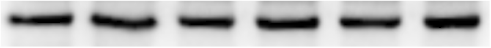


**Poly(I:C) (10 ug/ml)**

**LPS (10 ug/ml)**

**YQPC (4 ug/ml)**

**- - - - + +**

**- - + + - -**

**- + - + - +**

**P-P65(s536**)

**P65**

**a**
